# Confessions at the high and intensive care ward: what drives professionals in their daily practice?

**DOI:** 10.1192/j.eurpsy.2026.10390

**Published:** 2026-07-31

**Authors:** I. D. Jong

**Affiliations:** Ethics, Law & Humanities, Amsterdam University Medical Centers, Amsterdam, Netherlands

## Abstract

**Introduction:**

In the Netherlands, the High and Intensive Care model has been developed to enhance the quality of care and reduce coercion on acute psychiatric wards, with staff recognized as a crucial factor in its successful implementation. However, high staff turnover threatens both sustainable implementation of the model and the well-being of patients and healthcare professionals.

**Objectives:**

To inform strategies for staff retention and recruitment, this study explores the motivations of professionals working on High and Intensive Care wards.

**Methods:**

We used an innovative arts-based method known as the “confession booth” to collect data that uncovers deep motivations and encourages individuals to reflect anonymously. The confession booth, a designated room on the ward, invited staff to send voice messages via WhatsApp, in response to three prompts: 1) What impressed them most during their shift 2) What made them most proud, and 3) What frustrated them most. The confession booth operated simultaneously across eight High and Intensive Care wards in the Netherlands over the course of two weeks.

**Results:**

Thematic analysis identified four overarching themes, each comprising multiple subthemes: meaningful engagement, professional agency, collaborative competence, and security. Participants emphasized that High and Intensive Care work is driven by a deep sense of compassion and a strong commitment to supporting patient recovery. The complexity of the work, along with active involvement in clinical decision-making, was described as both meaningful and professionally rewarding. Effective teamwork, particularly within a flexible, skilled, familiar team, was seen as essential to both job satisfaction and safety. Feeling safe emerged as a crucial prerequisite for all aspects of the work in which effective collaboration and adequate support from law enforcement were essential to maintaining this safety.

**Conclusions:**

To safeguard care quality, the persistent vicious cycle of insufficient attention to staff retention must be broken through structural investment in well-being, recognition, and reward. Retention is shaped by meaningful work, opportunities to apply expertise, effective teamwork, and safe working conditions. The confession booth provided a flexible and anonymous space for reflection, offering valuable insights. These findings present a strong foundation for improving retention and recruitment within High and Intensive Care settings.

**Disclosure of Interest:**

None Declared

